# Combining Serum DNA Methylation Biomarkers and Protein Tumor Markers Improved Clinical Sensitivity for Early Detection of Colorectal Cancer

**DOI:** 10.1155/2021/6613987

**Published:** 2021-04-21

**Authors:** Guoying Zhang, Fang He, Guodong Zhao, Zihui Huang, Xiang Li, Xia Xia, Yidi Guo, Weiping Xu, Shangmin Xiong, Yong Ma, Minxue Zheng, Wanli Liu

**Affiliations:** ^1^Department of Clinical Laboratory, Nanjing Integrated Traditional Chinese and Western Medicine Hospital, Nanjing, Jiangsu 210014, China; ^2^Zhejiang University Kunshan Biotechnology Laboratory, Zhejiang University Kunshan Innovation Institute, Kunshan, Jiangsu 215300, China; ^3^Suzhou VersaBio Technologies Co. Ltd., Kunshan Jiangsu 215300, China; ^4^Department of Clinical Laboratory, Nanjing Traditional Chinese Medicine Hospital, Nanjing, Jiangsu, China; ^5^Suzhou Institute of Biomedical Engineering and Technology, Chinese Academy of Sciences, Suzhou Jiangsu 215163, China

## Abstract

**Background:**

Colorectal cancer (CRC) is one of the leading causes of cancer deaths worldwide and in China. Early CRC screening is the best approach to reduce its incidence and mortality rates. The ColoDefense test, a multiplex qPCR assay simultaneously detecting both methylated *SEPT9* and *SDC2* genes, has demonstrated improved clinical performance on either methylation biomarker alone for CRC screening with both blood and stool samples.

**Method:**

Leftover blood chemistry test samples from 125 CRC, 35 advanced adenoma, and 35 small polyp patients and 92 healthy control subjects were examined by the ColoDefense test. Among these samples, the levels of three circulating tumor markers, CEA, AFP, and CA19-9, were also measured for 106 CRC, 28 advanced adenoma, and 20 small polyp patients and all control subjects.

**Results:**

Due to the smaller volume and extended storage in nonfrozen state, the ColoDefense test with these samples exhibited reduced performance for all stages of CRC and advanced adenomas. The performance of CEA, AFP, and CA19-9 and their various combinations was also evaluated for CRC screening to identify the tumor marker combinations with the best performance. When combined with the ColoDefense test, the identified combinations did improve the clinical performance.

**Conclusion:**

These results suggested a rational path towards developing a CRC screening method that takes advantage of leftover blood chemistry test samples. The successful development of such a method will undoubtedly help promote early CRC screening by increasing its accessibility for the general public.

## 1. Introduction

According to the most recent statistics, colorectal cancer (CRC) ranked the third for incidence rate and the second for mortality rate among all cancers worldwide in 2018 [1]. The rankings for incidence and mortality rates of CRC in China were the second and the fifth, respectively, based on the same study. Moreover, due to changes towards a more westernized lifestyle brought on by fast advancing economy, Chinese CRC incidence rate has seen steady increase in the recent years [2].

Early detection of CRC and precancerous lesions is the proven approach to reduce human suffering and economic hardship caused by CRC [3]. However, due to its high cost, limited accessibility, and associated inconvenience, colonoscopy, the gold standard of CRC screening, is hardly a primary CRC screening method and has not been widely accepted by the Chinese population [4]. On the other hand, fecal immunochemical test (FIT) and guaiac fecal occult blood test (gFOBT), though cheaper and more convenient, have rather low diagnostic accuracy, especially for precancerous lesions and early-stage CRCs [5, 6].

Therefore, a low-cost, convenient, and more accurate screening method will go a long way to promote early CRC screening in China. The ColoDefense test that we have recently developed just may become such an alternative [7–9]. It is a multiplex quantitative PCR (qPCR) assay simultaneously detecting methylated DNA biomarkers within *septin 9* (*SEPT9*) and *syndecan 2* (*SDC2*) genes. Aberrant *SEPT9* methylation was the biomarker targeted by Epi proColon 2.0 test, a FDA-approved CRC screening assay detecting methylated *SEPT9* (*mSEPT9*) in blood [10, 11]. And *SDC2* has been shown to be hypermethylated in feces and blood of most CRC patients [12, 13]. The sensitivities of blood ColoDefense test for the detection of advanced adenoma (AA), stage I CRC, and all CRC were 47.8%, 80.0%, and 88.9%, respectively, significantly higher than those of FIT and gFOBT tests [14]. They were also higher than those of *mSEPT9* or methylated *SDC2* (*mSDC2*) alone. In addition, when stool samples were used instead, the sensitivity of the ColoDefense test was further improved to 66.7% for AA detection without any significant impact on its specificity of 91.9%, an enhanced advantage for CRC prevention [8].

Like Epi proColon 2.0 test, the standard input sample size of 10 mL peripheral blood for the ColoDefense test would be depleted after a single test, leaving no room for any error during sample processing. In case error did occur, additional blood draw would be required to repeat the test, which would not only be more costly but also logistically inconvenient for both the patient and the medical personnel. On the other hand, routine blood chemistry tests have been widely employed for disease diagnoses and regular physical examinations in the clinics, and there are always residual samples left after the tests. Instead of being discarded, if such leftover samples can be used, it will not only eliminate the requirement of a blood draw specific for CRC screening and the associated cost but also help to make such a powerful tool for CRC prevention more accessible for the general public, especially in developing countries like China.

However, using leftover samples of significantly smaller volumes for the ColoDefense test might reduce the clinical sensitivity of the assay [9]. One possible approach to compensate for such a potential adverse effect was to incorporate additional biomarkers in the screening test. One obvious group of candidate biomarkers for this purpose was serum tumor markers [15] as demonstrated by the CancerSEEK test [16]. By combining tumor markers and drive gene mutations in peripheral circulation, the CancerSEEK test improved the detection and localization of various cancers. Alpha-fetoprotein (AFP) has been mostly used as a tumor marker for hepatocellular carcinoma [17], though it has also been suggested as a marker for CRC [18]. In addition to its primary use as a tumor marker for CRC [19, 20], elevated serum carcinoembryonic antigen (CEA) level has also been implicated in various other cancers including breast [21, 22], lung [23], and gastric [24, 25] cancers. Similarly, while mostly used as a tumor marker for pancreatic cancer [26, 27], carbohydrate antigen 19-9 (CA19-9) has also been suggested as a tumor marker for ovarian cancer [28] as well as CRC [29–31]. Thus, the aims of this study are to evaluate the clinical performance of the ColoDefense test with a small sample volume of 1 mL and to investigate whether adding AFP, CEA, and CA19-9 measurements can improve that performance.

## 2. Materials and Methods

### 2.1. Sample Collection

Serum specimens were collected from 125 CRC (3 stage 0, 19 stage I, 38 stage II, 28 stage III, and 37 stage IV), 35 AA (≥1 cm in the greatest dimension or with high-grade dysplasia or with ≥25% villous histologic features), and 35 small polyp (SP, <1 cm in the greatest dimension or without high-grade dysplasia or villous component) patients and 92 colonoscopy-negative control subjects at Nanjing Integrated Traditional Chinese and Western Medicine Hospital. The diagnoses of all patients were made by colonoscopy and histologically confirmed by a pathologist. Ten milliliter blood was drawn from each subject, and the serum fractions were immediately isolated by centrifugation at 600 g for 15 minutes for blood biochemistry test. The remaining serum samples after the test were stored at 20 to 25°C for up to 10 hours, followed by 4°C storage for no more than 48 hours before being transferred to -80°C for long-term preservation and storage. The study was approved by the Institutional Review Board of Nanjing Integrated Traditional Chinese and Western Medicine Hospital (Ethics Committee reference number [2018]13), and the informed consent was obtained from all participating patients and control subjects.

### 2.2. DNA Extraction, Bisulfite Treatment, and Methylation-Specific Quantitative PCR

Circulating free DNA (cfDNA) from the serum samples was extracted using a cfDNA extraction kit (Suzhou VersaBio Technologies Co. Ltd., Kunshan, Jiangsu, China) from 1 mL remaining serum and eluted with 100 *μ*L elution buffer. The 100 *μ*L purified cfDNA then underwent bisulfite conversion, and the converted DNA was purified into 60 *μ*L elution buffer using a bisulfite conversion kit (Suzhou VersaBio Technologies Co. Ltd.). Purified DNA was examined by the ColoDefense test (Suzhou VersaBio Technologies Co. Ltd.), a methylation-specific quantitative PCR (qPCR) simultaneously detecting *mSEPT9*, *mSDC2*, and internal *beta-actin* (*ACTB*) control in a single multiplex qPCR reaction [14]. Three qPCR replicates were performed for each sample. The reaction volume was 30 *μ*L with 15 *μ*L DNA and 15 *μ*L PCR master mix. Real-time PCR was performed on an LC480-II thermal cycler (Roche Diagnostics, Pleasanton, California, USA) using the following cycling conditions: activation at 95°C for 30 minutes, 50 cycles of 95°C for 10 seconds, 58°C for 30 seconds, 72°C for 10 seconds, and final cooling to 40°C for 30 seconds. Both kits and the instrument were used according to the manufacturer's instructions.

### 2.3. Serum Tumor Marker Detection

Serum CEA, AFP, and CA19-9 levels were measured by the corresponding detection kits (Cat. No.: 09788458, 03305838, and 10491244, Siemens Healthcare Diagnostics Inc., Tarrytown, New York, USA) on an ADVIA Centaur XP Immunoassay System (Siemens Healthcare GmbH, Erlangen, Germany) at the Department of Clinical Laboratory of Nanjing Integrated Traditional Chinese and Western Medicine Hospital. All kits and the instrument were used according to the manufacturer's instructions. The normal reference values were as follows: CEA ≤ 9.8 ng/mL, AFP ≤ 10 U/mL, and CA19‐9 ≤ 37 U/mL.

### 2.4. Data Analysis

The scoring schemes for *mSEPT9* alone, *mSDC2* alone, and the combined ColoDefense test were the same as in Zhao et al. [14]. Specifically, the qPCR results were considered “invalid” if *ACTB* Cp was greater than 35.0, and *mSEPT9* and *mSDC2* were “detected” if their Cp values were less than 45.0 and 50.0, respectively. *mSEPT9* was analyzed by using a 1/3 rule in which a serum sample was scored positive if one of the three PCR replicates had a valid amplification curve (1/3 algorithm). And *mSDC2* was analyzed by using a 2/3 rule, whereby to be called positive, two of the three PCR replicates must have valid amplification curves (2/3 algorithm). The serum sample would be considered as positive if either *mSEPT9* or *mSDC2* was positive. Data were subjected to statistical analysis by IBM SPSS for Windows version 22.0. The Pearson chi-square test was used to determine the relationship between two categorical variables at the significance level of *p* < 0.05 according to the following categorization scheme: for tumor stage, 0 for AA, 1 for combined stages 0 and I as there were only 3 stage 0 samples, 2 for stage II, 3 for stage III, and 4 for stage IV; for age, 2 for 20-29, 3 for 30-39 until 9 for ≥90 years old; for gender, M for male and F for female; for tumor location, P for proximal, D for distal, and R for rectal tumors; for tumor size, 0 for 0-0.99 cm, 1 for 1-1.99 cm until 10 for ≥10 cm in the largest dimension; for biomarker result, 1 for positive and 0 for negative. Receiver operating characteristic (ROC) curves were plotted using the following scoring scheme: for subject type, 1 for SP, AA, or CRC patients and 0 for control subjects; for *mSEPT9* or *mSDC2* result, 1 for positive and 0 for negative.

## 3. Results

To evaluate the feasibility of using minimal amounts of leftover serum samples from routine blood biochemistry test for CRC screening by the ColoDefense test and compare its performance for early CRC screening to that of serum tumor markers, such samples were collected from 125 CRC patients at Nanjing Integrated Traditional Chinese and Western Medicine Hospital, including 3 stage 0, 19 stage I, 38 stage II, 28 stage III, and 37 stage IV patients (Table 1). In addition, similar leftover serum samples were also collected from 35 AA patients, 35 SP patients, and 92 healthy control subjects. The CRC patients ranged from 24 to 90 years old with a median age of 60. The age of AA patients ranged from 29 to 74, the range of SP patient age was from 25 to 78, and the median age of both groups was 57. The control subjects ranged from 18 to 68 years old, whose median age was 36.5. Male subjects accounted for 63.2%, 54.3%, 62.9%, and 34.8% of CRC, AA, and SP patients and control group, respectively.

**Figure 1:**
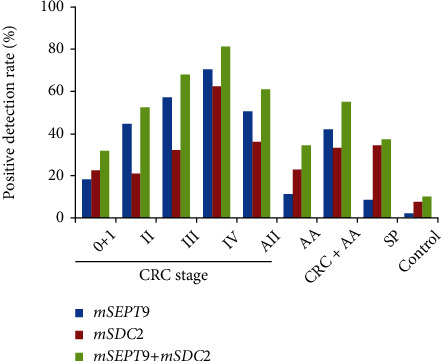
Positive detection rates of *mSEPT9*, *mSDC2*, and combined ColoDefense tests for detecting different disease states.

Consistent with our earlier observations [14], combining both *mSEPT9* and *mSDC2* showed higher sensitivities than either *mSEPT9* alone or *mSDC2* alone for all disease states (Figure 1 and Supplementary Table 1). For example, the sensitivities of *mSEPT9*, *mSDC2*, and the two-biomarker combination (*mSEPT9+mSDC2*) for CRC detection were 50.4%, 36.0%, and 60.8% with specificities of 97.8%, 92.4%, and 90.2%, respectively, whereas their sensitivities for AA detection were 11.4%, 22.9%, and 34.3%. More careful examination revealed that the sensitivities of *mSEPT9* and *mSDC2* for different disease states along the spectrum of malignancy displayed different patterns. The sensitivities of *mSEPT9* increased incrementally from 11.4% for AA to 70.3% for stage IV CRC. In contrast, the sensitivities of *mSDC2* remained rather constant at 21-23% for AA and stage 0 to II CRC and then increased to 62.2% for stage IV CRC. In addition, the sensitivities of *mSDC2* were higher than those of *mSEPT9* from AA to stage I CRC but lower for stage II to IV CRC. More strikingly, whereas the sensitivity of *mSEPT9* was merely 8.6%, that of *mSDC2* was significantly higher at 34.3%.

**Figure 2:**
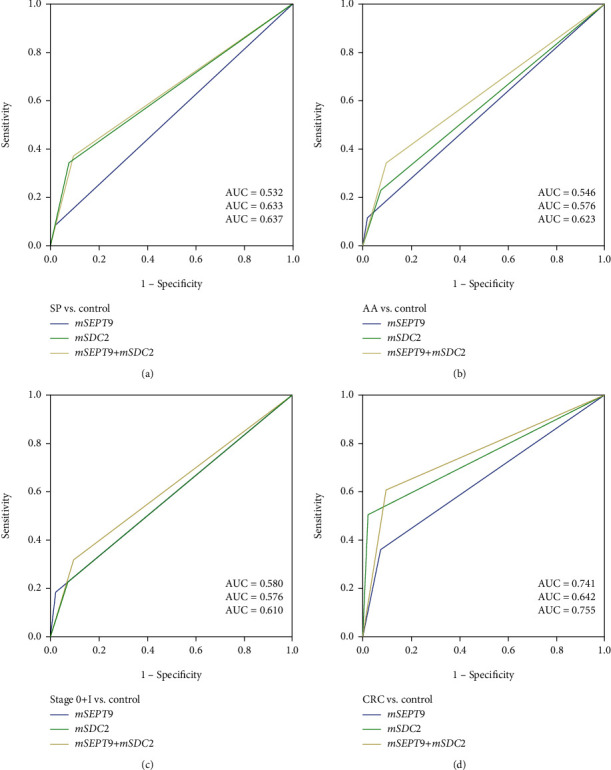
ROC curves for ColoDefense test in detecting (a) SP, (b) AA, (c) stage 0 and I CRC, and (d) all CRC.

To further differentiate the performance of *mSEPT9*, *mSDC2*, and *mSEPT9+mSDC2*, ROC curves for detecting different disease states were generated (Figure 2). The results demonstrated a similar trend to that of sensitivities. For SP, AA, early CRC (stages 0 and I), and all CRC groups, *mSEPT9+mSDC2* produced the largest area under the curve (AUC) values. For precancerous disease states, SP and AA, AUC values of *mSDC2* were larger than those of *mSEPT9*, 0.633 (95% CI: 0.517-0.750) vs. 0.532 (95% CI: 0.417-0.647) for SP and 0.576 (95% CI: 0.459-0.693) vs. 0.546 (95% CI: 0.430-0.663) for AA. For early CRC including stage 0 and I cancers, the AUC value of *mSDC2*, 0.576 (95% CI: 0.434-0.717), was nearly identical to that of *mSEPT9*, 0.580 (95% CI: 0.437-0.723). On the contrary, for all CRCs, the AUC value of *mSEPT9*, 0.741 (95% CI: 0.676-0.806), was larger than that of *mSDC2*, 0.642 (95% CI: 0.569-0.715) instead. Furthermore, based on the Pearson chi-square test, there was no significant correlation between the positive detection rates of *mSEPT9*, *mSDC2*, and *mSEPT9+mSDC2* and age, gender, tumor location, and tumor size (*p* > 0.05, Table 2), mostly consistent with earlier observations [9, 14].

**Figure 3:**
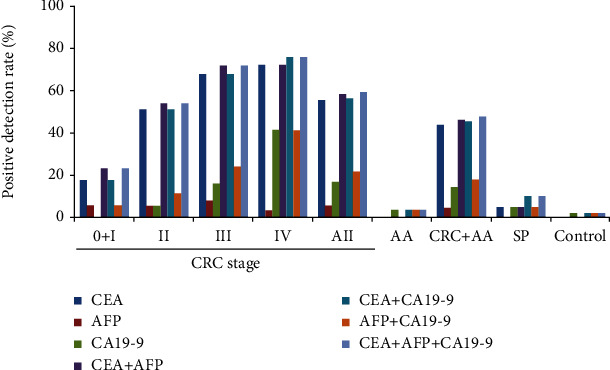
Positive detection rates of CEA, AFP, and CA19-9 and their various combinations for detecting different disease states.

Among all enrolled subjects, serum CEA, AFP, and CA19-9 levels were measured in 2 out of 3 stage 0, 15 out of 19 stage I, 35 out of 38 stage II, 25 out of 28 stage III, 29 out of 37 stage IV CRC, 28 out of 35 AA, 20 out of 35 SP patients, and all control subjects. As a single biomarker, CEA demonstrated the highest sensitivities for all CRC stages, 17.6% for stages 0 and I combined, 51.4% for stage II, 68.0% for stage III, 72.4% for stage IV, and 55.7% for all CRCs with a specificity of 100.0% (Figure 3 and Supplementary Table 2). In contrast, the sensitivities of AFP for stage II, stage III, stage IV, and all CRCs, 5.7%, 8.0%, 3.4%, and 5.7%, respectively, were the lowest among the three tumor markers. Whereas the sensitivities of CA19-9 for stage 0 to II CRCs were similar to those of AFP, its sensitivities for stage III and IV CRCs were 16.0% and 41.4%, much higher than those of AFP. In comparison to the performance of single tumor markers, two-marker combinations of CEA plus AFP (CEA+AFP) and CEA plus CA19-9 (CEA+CA19-9) showed similar sensitivities to those of CEA alone for detecting all-stage CRCs, but much higher than those of AFP+CA19-9 combination. In addition, the sensitivities of three-marker combination (CEA+AFP+CA19-9) only marginally improved upon those of CEA+AFP, CEA+CA19-9, and CEA alone.

As the sample sizes were different between the data of DNA methylation biomarkers and those of tumor markers, we reassessed the sensitivities of *mSEPT9*, *mSDC2*, and *mSEPT9+mSDC2* for detecting the different disease states using only cases having tumor marker data (Supplementary Table 3). When compared to the tumor marker data, the most striking observation was that the sensitivities of *mSDC2* and *mSEPT9+mSDC2* for AA and SP detection were much higher than those of even the best tumor marker combinations, CEA+CA19-9 and CEA+AFP+CA19-9. Whereas the sensitivities of CEA+CA19-9 and CEA+AFP+CA19-9 were 3.6% and 10%, respectively, for AA and SP, those of *mSDC2* were 25% for both AA and SP, and those of *mSEPT9+mSDC2* were 39.3% and 30.0% for AA and SP, respectively, which were 10.9- and 3-fold higher than those of the two tumor marker combinations.

**Figure 4:**
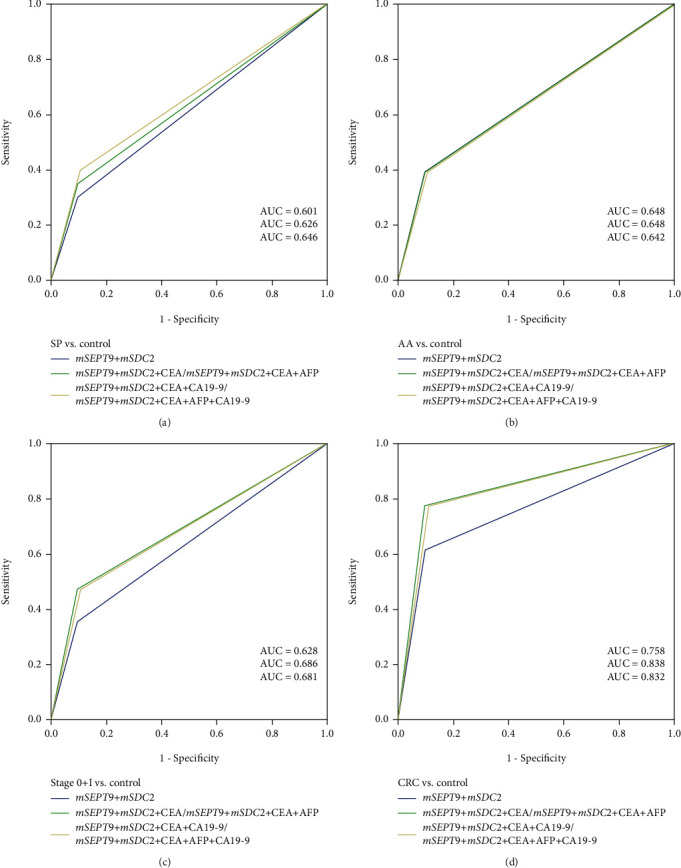
ROC curves for combined ColoDefense and tumor marker tests in detecting (a) SP, (b) AA, (c) stage 0 and I CRC, and (d) all CRC.

To investigate whether combining both DNA methylation biomarkers and tumor markers could further improve the sensitivities for SP, AA, and CRC detection, the DNA methylation biomarker combination with the highest sensitivities, *mSEPT9+mSDC2*, was further combined with the four tumor marker combinations having similar performance, CEA alone, CEA+AFP, CEA+CA19-9, and CEA+AFP+CA19-9. Indeed, in comparison to *mSEPT9+mSDC2*, all four combinations of DNA methylation biomarkers and tumor markers led to improved sensitivities for all stages of CRC, from 35.3% to 47.1% for stages 0 and I combined, from 48.6% to 74.3% for stage II, from 64.0% to 80.0% for stage III, from 89.7% to 96.6% for stage IV, and from 61.3% to 77.4% for all CRCs (Table 3). Consistent with the earlier observation that tumor markers showed very low sensitivities for AA and SP (Figure 3 and Supplementary Table 2), combining *mSEPT9+mSDC2* with any tumor marker combination did not improve the sensitivity for AA detection, and the improvement for SP detection was not significant taking into account the small sample size of the SP group. These results were further confirmed by ROC analysis, where the largest improvement occurred for CRC detection (Figure 4). The AUC value for CRC detection was improved from 0.758 for *mSEPT9+mSDC2* to between 0.83 and 0.84 for all four combinations of *mSEPT9+mSDC2* and tumor markers. On the other hand, adding tumor markers to *mSEPT9+mSDC2* did not show any improvement in the AUC value for AA detection.

## 4. Discussion

CRC is one of the leading causes of cancer deaths globally and constitutes serious burdens on the welfare of societies and individuals especially in developing countries like China. The best approach to lighten such burdens is to promote early CRC screening, which has been demonstrated to reduce CRC incidence and mortality rates. Despite its status as the gold standard for early CRC screening, colonoscopy has not been well received by the general public due to its high cost, burdensome preparation, potential complications, and limited accessibility in China.

Detection of cancer-related aberrant DNA methylation has been widely studied as a general approach for cancer screening and diagnosis [32]. And methylation of various genes has been proposed as biomarkers for CRC [33] including *SEPT9* [34, 35], *SDC2* [12], *secreted frizzled related protein 2* (*SFRP2*) [36, 37], *N-myc downstream regulated gene family member 4* (*NDRG4*) [37, 38], *vimentin* (*VIM*) [37, 39], and *bone morphogenetic protein 3* (*BMP3*) [38] genes. Among these, *SEPT9* and *SDC2* methylation for CRC detection has been studied most extensively. In case control studies, the sensitivity and specificity of blood *mSEPT9* for CRC detection ranged from 69 to 79% and 82 to 99%, respectively [40]. According to the same study, the pooled sensitivities of *mSEPT9* for AA and stage I CRC were 15% and 45%, respectively. The performance of *mSDC2* as biomarker for CRC detection has been studied with both blood and stool samples, where the sensitivities and the specificities ranged, respectively, from 80% to 90% and from 80% to 95% [12, 13, 41]. The sensitivities of *mSDC2* for early CRC and precancerous lesions appeared to be higher than those of *mSEPT9*, 83.3% and 92.3% for stage I CRC between two studies [12, 13], 58.2% for AA [41], 81.1% for adenomas [42], and 33.3% for SP [13]. We have recently developed a multiplex qPCR that simultaneously detects *mSEPT9* and *mSDC2*, the ColoDefense test. The performance of *mSEPT9* and *mSDC2* as a single blood biomarker was within the ranges observed in the previous studies [14]. The sensitivities of *mSEPT9* for AA, stage I CRC, and all CRC detection were 12.1%, 65.0%, and 82.1%, respectively, and those of *mSDC2* were 43.5%, 55.0%, and 69.2%. Combining both methylation biomarkers in the ColoDefense test improved the sensitivities to 47.8%, 80.0%, and 88.9%, respectively, for AA, stage I CRC, and all CRC detection, which were on par with the clinical performance of Cologuard, another FDA-approved CRC screening test with 42.4% and 92.3% sensitivities, respectively, for AA and CRC detection [43]. When stool samples were used, the sensitivities of *mSEPT9* for AA and CRC detection were 50.0% and 79.8%, respectively, and those of *mSDC2* were 50.0% and 85.1% [8]. When the two methylation biomarkers were combined in the ColoDefense test, the sensitivities were improved to 66.7% and 90.4%, respectively. Notably, the sensitivity of the ColoDefense test for AA detection was much higher with stool samples than that with blood samples.

Routine blood chemistry tests are performed extensively in clinics and always have leftover blood samples after the tests. If such leftover samples can be used, it will help increase the accessibility to early CRC screening for the general public. We have previously examined the performance of the ColoDefense test with such samples [9]. Whereas smaller input volumes appeared to only reduce the sensitivities of the ColoDefense test for stage I CRC detection, the impact for AA detection was not known. In addition, leftover samples from blood chemistry tests are routinely left in nonfrozen state. As prolonged storage in nonfrozen state has been shown to lead to ctDNA loss [44], such an effect may further diminish the performance for detecting early-stage CRC and precancerous lesions.

Thus, we have expanded the scope of the study in this work to further define the impact of reduced sample size and prolonged storage in nonfrozen state for early CRC screening. For the regular ColoDefense test with 10 mL input sample size and 4°C storage, the sensitivities for the detection of SP, AA, and stage I to IV CRC were 23.1%, 47.8%, 80.0%, 90.0%, 89.5%, and 100%, respectively [14]. Indeed, with the exception of SP, the sensitivities of the ColoDefense test for the detection of AA and stage I to IV CRC were reduced to 34.3%, 31.8%, 52.6%, 67.9%, and 81.1% in this study (Figure 1 and Supplementary Table 1). In an attempt to compensate for such a loss, the circulating levels of three tumor markers, CEA, AFP, and CA19-9, were examined (Figure 3 and Supplementary Table 2). As a single biomarker, CEA appeared to be the best one for detecting all stages of CRC, whereas AFP showed the worst performance. And any two-biomarker or three-biomarker combination including CEA only marginally enhanced CEA performance. Furthermore, the sensitivities for SP and AA of any single biomarker or biomarker combinations were rather minimal. The results suggested that more tumor markers need to be tested to identify the one(s) that may provide additive or even synergistic effect on CEA performance. Lastly, when the ColoDefense test was further combined with CEA alone or three different combinations of CEA, AFP, and CA19-9, the sensitivities for detecting stage 0+I, II, III, and IV CRC were improved from 35.3%, 48.6%, 64.0%, and 89.7% to 47.1%, 74.3%, 80.0%, and 96.6%, respectively (Table 3), though still not reaching the performance level of regular ColoDefense test. Moreover, adding tumor markers to ColoDefense test did not improve the sensitivity for AA detection.

Our results indicated that more studies are needed to further improve upon ColoDefense performance with leftover samples from blood biochemistry tests to make it comparable to regular ColoDefense test. Such approaches could include incorporating additional biomarkers and simply storing leftover samples immediately at 4°C. In addition, even though no two-tumor-marker and three-tumor-marker combination including CEA showed much improvement on CEA performance, it would be premature to eliminate such options as the lack of meaningful improvement could be an artifact of relatively small sample size of this study. For example, enrollment of more research subjects may help differentiate the effect of adding CA19-9 on CEA performance.

## 5. Conclusion

In this study, we have demonstrated that adding tumor markers to the ColoDefense test with leftover samples from blood chemistry tests could improve its performance on CRC detection. Even though the improved performance did not reach the level of regular ColoDefense test, the results did suggest that adding additional biomarkers could be an approach to achieve that goal. The successful development of a screening method taking advantage of leftover blood chemistry test samples will undoubtedly help promote early CRC screening and prevention by increasing its accessibility for the general public, especially for countries and regions with limited resources.

## Figures and Tables

**Table 1:** Characteristics of subjects enrolled in this study.

	Number (*N*)	Gender		Age			
		Male [*n* (%)]	Female [*n* (%)]	Min	Max	Mean	Median
CRC stage							
0	3	3 (100.0%)	0 (0.0%)	52	61	56.3	56
I	19	11 (57.9%)	8 (42.1%)	42	83	63.9	61
II	38	23 (60.5%)	15 (39.5%)	24	90	59.8	57
III	28	19 (67.9%)	9 (32.1%)	35	83	61.6	59.5
IV	37	23 (62.2%)	14 (37.8%)	36	83	60.4	62
0 and I	22	14 (63.6%)	8 (36.4%)	42	83	62.9	60.5
All	125	79 (63.2%)	46 (36.8%)	24	90	60.9	60
AA	35	19 (54.3%)	16 (45.7%)	29	74	56.5	57
CRC and AA	160	98 (61.3%)	62 (38.8%)	24	90	60.0	59
SP	35	22 (62.9%)	13 (37.1%)	25	78	55.2	57
Control	92	32 (34.8%)	60 (65.2%)	18	68	37.3	36.5

**Table 2:** Positive detection rates of ColoDefense test for CRC of different ages, genders, tumor locations, and tumor sizes.

	Number (*N*)	*mSEPT9*			*mSDC2*			*mSEPT9*+*mSDC2*		
		Positive [*n* (%)]	Chi-square test		Positive [*n* (%)]	Chi-square test		Positive [*n* (%)]	Chi-square test	
			*Χ* ^2^	*p*		*Χ* ^2^	*p*		*Χ* ^2^	*p*
Age										
20-39	5	2 (40.0)	0.40	0.941	1 (20.0)	4.74	0.192	3 (60.0)	4.94	0.176
40-59	57	28 (49.1)			18 (31.6)			30 (52.6)		
60-79	54	28 (51.9)			20 (37.0)			35 (64.8)		
≥80	9	5 (55.6)			6 (66.7)			8 (88.9)		

Gender										
Male	79	43 (54.4)	1.40	0.238	27 (34.2)	0.31	0.578	49 (62)	0.14	0.713
Female	46	20 (43.5)			18 (39.1)			27 (58.7)		

Tumor location										
Proximal	23	11 (47.8)	0.00	0.999	9 (39.1)	1.50	0.473	14 (60.9)	0.02	0.992
Distal	23	11 (47.8)			6 (26.1)			14 (60.9)		
Rectal	57	27 (47.4)			23 (40.4)			34 (59.6)		
N/A	22	14 (63.6)	/	/	7 (31.8)	/	/	14 (63.6)	/	/

Tumor size										
<3 cm	24	12 (50.0)	0.33	0.847	8 (33.3)	0.23	0.890	13 (54.2)	0.23	0.893
3-6 cm	74	34 (45.9)			25 (33.8)			44 (59.5)		
≥6 cm	15	8 (53.3)			6 (40.0)			9 (60.0)		
N/A	12	9 (75.0)	/	/	6 (50.0)	/	/	10 (83.3)	/	/

N/A: information not available. “/”: data not included in the Pearson chi-square test analysis.

**Table 3:** Performance of combining both DNA methylation biomarkers and tumor markers for detecting different disease states.

		*mSEPT9*+*mSDC2*	*mSEPT9*+*mSDC2*+CEA and *mSEPT9*+*mSDC2*+CEA+AFP	*mSEPT9*+*mSDC2*+CEA+CA19-9 and *mSEPT9*+*mSDC2*+CEA+AFP+CA19-9
Sensitivity (95% CI)	CRC stage			
	0+I	35.3 (15.3-61.4)	47.1 (23.9-71.5)	
	II	48.6 (31.7-65.7)	74.3 (56.4-86.9)	
	III	64.0 (42.6-81.3)	80.0 (58.7-92.4)	
	IV	89.7 (71.5-97.3)	96.6 (80.4-99.8)	
	All	61.3 (51.3-70.5)	77.4 (68.0-84.7)	
	AA	39.3 (22.1-59.3)	39.3 (22.1-59.3)	
	CRC+AA	56.7 (47.9-65.2)	69.4 (60.8-76.9)	
	SP	30.0 (12.8-54.3)	35.0 (16.3-59.1)	40.0 (20.0-63.6)
Specificity (95% CI)		90.2 (81.8-95.2)	90.2 (81.8-95.2)	89.1 (80.5-94.4)

## Data Availability

The datasets used and/or analyzed during the current study are available from the corresponding author on reasonable request.

## Supplementary Materials

Supplementary Materials Supplementary Table 1: performance of ColoDefense test for detecting different disease states. Supplementary Table 2: performance of CEA, AFP, and CA19-9 for detecting different disease states. Supplementary Table 3: performance of ColoDefense test in detecting different disease states for the subjects who also had CEA, AFP, and CA19-9 measurements.

## References

[1] Bray F., Ferlay J., Soerjomataram I., Siegel R. L., Torre L. A., Jemal A. (2018). Global cancer statistics 2018: GLOBOCAN estimates of incidence and mortality worldwide for 36 cancers in 185 countries. *CA: a Cancer Journal for Clinicians*.

[2] Chen W., Zheng R., Baade P. D. (2016). Cancer statistics in China, 2015. *CA: a Cancer Journal for Clinicians*.

[3] Vatandoost N., Ghanbari J., Mojaver M. (2016). Early detection of colorectal cancer: from conventional methods to novel biomarkers. *Journal of Cancer Research and Clinical Oncology*.

[4] Chen H., Li N., Ren J. (2019). Participation and yield of a population-based colorectal cancer screening programme in China. *Gut*.

[5] Imperiale T. F., Gruber R. N., Stump T. E., Emmett T. W., Monahan P. O. (2019). Performance characteristics of fecal immunochemical tests for colorectal cancer and advanced adenomatous polyps: a systematic review and meta-analysis. *Annals of Internal Medicine*.

[6] Tinmouth J., Lansdorp-Vogelaar I., Allison J. E. (2015). Faecal immunochemical tests versus guaiac faecal occult blood tests: what clinicians and colorectal cancer screening programme organisers need to know. *Gut*.

[7] Zhao G., Tian Y., Du Y. (2019). Comparison of CerviHPV and hybrid capture 2 HPV tests for detection of high-risk HPV infection in cervical swab specimens. *Diagnostic Cytopathology*.

[8] Zhao G., Liu X., Liu Y. (2020). Aberrant DNA methylation of SEPT9 and SDC2 in stool specimens as an integrated biomarker for colorectal cancer early detection. *Frontiers in Genetics*.

[9] Chen Y., Wang Z., Zhao G. (2019). Performance of a novel blood-based early colorectal cancer screening assay in remaining serum after the blood biochemical test. *Disease Markers*.

[10] Lamb Y. N., Dhillon S. (2017). Epi proColon® 2.0 CE: a blood-based screening test for colorectal cancer. *Molecular Diagnosis & Therapy*.

[11] Worm Orntoft M. B. (2018). Review of blood-based colorectal cancer screening: how far are circulating cell-free DNA methylation markers from clinical implementation?. *Clinical Colorectal Cancer*.

[12] Oh T., Kim N., Moon Y. (2013). Genome-wide identification and validation of a novel methylation biomarker, SDC2, for blood-based detection of colorectal cancer. *The Journal of Molecular Diagnostics*.

[13] Oh T. J., Oh H. I., Seo Y. Y. (2017). Feasibility of quantifying SDC2 methylation in stool DNA for early detection of colorectal cancer. *Clinical Epigenetics*.

[14] Zhao G. D., Li H., Yang Z. X. (2019). Multiplex methylated DNA testing in plasma with high sensitivity and specificity for colorectal cancer screening. *Cancer medicine*.

[15] Perkins G. L., Slater E. D., Sanders G. K., Prichard J. G. (2003). Serum tumor markers. *American Family Physician*.

[16] Cohen J. D., Li L., Wang Y. (2018). Detection and localization of surgically resectable cancers with a multi-analyte blood test. *Science*.

[17] Sauzay C., Petit A., Bourgeois A. M. (2016). Alpha-foetoprotein (AFP): a multi-purpose marker in hepatocellular carcinoma. *Clinica Chimica Acta*.

[18] Anzai H., Kazama S., Kiyomatsu T. (2015). Alpha-fetoprotein-producing early rectal carcinoma: a rare case report and review. *World Journal of Surgical Oncology*.

[19] Nicholson B. D., Shinkins B., Pathiraja I. (2015). Blood CEA levels for detecting recurrent colorectal cancer. *Cochrane Database of Systematic Reviews*.

[20] Zhang S. Y., Lin M., Zhang H. B. (2015). Diagnostic value of carcinoembryonic antigen and carcinoma antigen 19-9 for colorectal carcinoma. *International Journal of Clinical and Experimental Pathology*.

[21] Shao Y., Sun X., He Y., Liu C., Liu H. (2015). Elevated levels of serum tumor markers CEA and CA15-3 are prognostic parameters for different molecular subtypes of breast cancer. *PLoS One*.

[22] Li X., Dai D., Chen B., Tang H., Xie X., Wei W. (2018). Clinicopathological and prognostic significance of cancer antigen 15-3 and carcinoembryonic antigen in breast cancer: a meta-analysis including 12,993 patients. *Disease Markers*.

[23] Arrieta O., Villarreal-Garza C., Martínez-Barrera L. (2013). Usefulness of serum carcinoembryonic antigen (CEA) in evaluating response to chemotherapy in patients with advanced non small-cell lung cancer: a prospective cohort study. *BMC Cancer*.

[24] Jin Z., Jiang W., Wang L. (2015). Biomarkers for gastric cancer: progression in early diagnosis and prognosis (review). *Oncology Letters*.

[25] Yu J., Zheng W. (2018). An alternative method for screening gastric cancer based on serum levels of CEA, CA19-9, and CA72-4. *Journal of Gastrointestinal Cancer*.

[26] Ballehaninna U. K., Chamberlain R. S. (2011). Serum CA 19-9 as a biomarker for pancreatic cancer-a comprehensive review. *Indian Journal of Surgical Oncology*.

[27] Goonetilleke K. S., Siriwardena A. K. (2007). Systematic review of carbohydrate antigen (CA 19-9) as a biochemical marker in the diagnosis of pancreatic cancer. *European Journal of Surgical Oncology*.

[28] Canney P. A., Wilkinson P. M., James R. D., Moore M. (1985). CA19-9 as a marker for ovarian cancer: alone and in comparison with CA125. *British Journal of Cancer*.

[29] Gao Y., Wang J., Zhou Y., Sheng S., Qian S. Y., Huo X. (2018). Evaluation of serum CEA, CA19-9, CA72-4, CA125 and ferritin as diagnostic markers and factors of clinical parameters for colorectal cancer. *Scientific Reports*.

[30] Yan J. X., Wang L. N., Wang L. N. (2014). The diagnostic value of serum carcino-embryonic antigen, alpha fetoprotein and carbohydrate antigen 19-9 for colorectal cancer. *Journal of Cancer Research and Therapeutics*.

[31] Zhang D., Yu M., Xu T., Xiong B. (2013). Predictive value of serum CEA, CA19-9 and CA125 in diagnosis of colorectal liver metastasis in Chinese population. *Hepato-Gastroenterology*.

[32] Mikeska T., Craig J. M. (2014). DNA methylation biomarkers: cancer and beyond. *Genes (Basel)*.

[33] Lam K., Pan K., Linnekamp J. F., Medema J. P., Kandimalla R. (2016). DNA methylation based biomarkers in colorectal cancer: a systematic review. *Biochimica et Biophysica Acta*.

[34] Lofton-Day C., Model F., DeVos T. (2008). DNA methylation biomarkers for blood-based colorectal cancer screening. *Clinical Chemistry*.

[35] Devos T., Tetzner R., Model F. (2009). Circulating methylated SEPT9 DNA in plasma is a biomarker for colorectal cancer. *Clinical Chemistry*.

[36] Yang Q., Huang T., Ye G., Wang B., Zhang X. (2016). Methylation of *SFRP2* gene as a promising noninvasive biomarker using feces in colorectal cancer diagnosis: a systematic meta-analysis. *Scientific Reports*.

[37] Lu H., Huang S., Zhang X. (2014). DNA methylation analysis of SFRP2, GATA4/5, NDRG4 and VIM for the detection of colorectal cancer in fecal DNA. *Oncology Letters*.

[38] Kisiel J. B., Yab T. C., Taylor W. R., Mahoney D. W., Ahlquist D. A. (2014). Stool methylated DNA markers decrease following colorectal cancer resection--implications for surveillance. *Digestive Diseases and Sciences*.

[39] Zou H., Harrington J. J., Shire A. M. (2007). Highly methylated genes in colorectal neoplasia: implications for screening. *Cancer Epidemiology, Biomarkers & Prevention*.

[40] Nian J., Sun X., Ming S. (2017). Diagnostic accuracy of methylated SEPT9 for blood-based colorectal cancer detection: a systematic review and meta-analysis. *Clinical and Translational Gastroenterology*.

[41] Niu F., Wen J., Fu X. (2017). Stool DNA test of methylated *syndecan*-2 for the early detection of colorectal neoplasia. *Cancer Epidemiology, Biomarkers & Prevention*.

[42] Barták B. K., Kalmár A., Péterfia B. (2017). Colorectal adenoma and cancer detection based on altered methylation pattern of SFRP1, SFRP2, SDC2, and PRIMA1 in plasma samples. *Epigenetics*.

[43] Imperiale T. F., Ransohoff D. F., Itzkowitz S. H. (2014). Multitarget stool DNA testing for colorectal-cancer screening. *The New England Journal of Medicine*.

[44] Kang Q., Henry N. L., Paoletti C. (2016). Comparative analysis of circulating tumor DNA stability In K_3_EDTA, Streck, and CellSave blood collection tubes. *Clinical Biochemistry*.

