# Supplementary material for: Combining Serum DNA Methylation Biomarkers and Protein Tumor Markers Improved Clinical Sensitivity for Early Detection of Colorectal Cancer
Source: Int J Genomics. 2021 Apr 21;2021:6613987. doi: 10.1155/2021/6613987 (PMC8084680; doi:10.1155/2021/6613987)
Supplement: Supplementary Materials — Supplementary Table 1: performance of ColoDefense test for detecting different disease states. Supplementary Table 2: performance of CEA, AFP, and CA19-9 for detecting different disease states. Supplementary Table 3: performance of ColoDefense test in detecting different disease states for the subjects who also had CEA, AFP, and CA19-9 measurements. [file 6613987.f1.docx]

**Supplementary Table 1**. Performance of ColoDefence test for detecting different disease states.

|  | | ***mSEPT9*** | ***mSDC2*** | ***mSEPT9+mSDC2*** |
| --- | --- | --- | --- | --- |
| **Sensitivity**  **(95% CI)** | **CRC stage** |  |  |  |
|  | **0+I** | 18.2 (6.0 - 41.0) | 22.7 (8.7 - 45.8) | 31.8 (14.7 - 54.9) |
|  | **II** | 44.7 (29.0 - 61.5) | 21.1 (10.1 - 37.8) | 52.6 (36.0 - 68.7) |
|  | **III** | 57.1 (37.4 - 75.0) | 32.1 (16.6 - 52.4) | 67.9 (47.6 - 83.4) |
|  | **IV** | 70.3 (52.8 - 83.6) | 62.2 (44.8 - 77.1) | 81.1 (64.3 - 91.4) |
|  | **All** | 50.4 (41.4 - 59.4) | 36.0 (27.8 - 45.1) | 60.8 (51.6 - 69.3) |
|  | **AA** | 11.4 (3.7 - 27.7) | 22.9 (11.0 - 40.6) | 34.3 (19.7 - 52.3) |
|  | **CRC + AA** | 41.9 (34.2 - 49.9) | 33.1 (26.0 - 41.1) | 55.0 (47.0 - 62.8) |
|  | **SP** | 8.6 (2.2 - 24.2) | 34.3 (19.7 - 52.3) | 37.1 (22.0 - 55.1) |
| **Specificity (95% CI)** | | 97.8 (91.6 - 99.6) | 92.4 (84.4 - 96.6) | 90.2 (81.8 - 95.2) |

**Supplementary Table 2**. Performance of CEA, AFP and CA19-9 for detecting different disease states.

| **Group** | | **Number**  **(N)** | **No. of positive/positive detection rate (95% CI)** | | | | | | |
| --- | --- | --- | --- | --- | --- | --- | --- | --- | --- |
|  |  |  | **CEA** | **AFP** | **CA19-9** | **CEA+AFP** | **CEA+**  **CA19-9** | **AFP+**  **CA19-9** | **CEA+AFP**  **+CA19-9** |
| **CRC stage** | |  |  |  |  |  |  |  |  |
|  | **0+I** | 17 | 3/17.6  (4.7-44.2) | 1/5.9  (0.3-30.7) | 0/0.0  (0.0-22.9) | 4/23.5  (7.8-50.2) | 3/17.6  (4.7-44.2) | 1/5.9  (0.3-30.8) | 4/23.5  (7.8-50.2) |
|  | **II** | 35 | 18/51.4  (34.3-68.3) | 2/5.7  (1.0-20.5) | 2/5.7  (1.0-20.5) | 19/54.3  (36.9-70.8) | 18/51.4  (34.3-68.3) | 4/11.4  (3.7-27.7) | 19/54.3  (36.9-70.8) |
|  | **III** | 25 | 17/68.0  (46.4-84.3) | 2/8.0  (1.4-27.5) | 4/16.0  (5.3-36.9) | 18/72.0  (50.4-87.1) | 17/68.0  (46.4-84.3) | 6/24.0  (10.2-45.5) | 18/72.0  (50.4-87.1) |
|  | **IV** | 29 | 21/72.4  (52.5-86.6) | 1/3.4  (0.2-19.6) | 12/41.4  (24.1-60.9) | 21/72.4  (52.5-86.6) | 22/75.9  (56.1-89.0) | 12/41.4  (24.1-60.9) | 22/75.9  (56.1-89.0) |
|  | **All** | 106 | 59/55.7  (45.7-65.2) | 6/5.7  (2.3-12.4) | 18/17.0  (10.6-25.8) | 62/58.5  (48.5-67.9) | 60/56.6  (46.6-66.1) | 23/21.7  (14.5-31.0) | 63/59.4  (49.4-68.7) |
| **AA** | | 28 | 0/0.0  (0.0-15.0) | 0/0.0  (0.0-15.0) | 1/3.6  (0.2-20.2) | 0/0.0  (0.0-15.0) | 1/3.6  (0.2-20.2) | 1/3.6  (0.2-20.2) | 1/3.6  (0.2-20.2) |
| **CRC + AA** | | 134 | 59/44.0  (35.6-52.9) | 6/4.5  (1.8-9.9) | 19/14.2  (9.0-21.5) | 62/46.3  (37.7-55.1) | 61/45.5  (37.0-54.3) | 24/17.9  (12.0-25.7) | 64/47.8  (39.1-56.5) |
| **SP** | | 20 | 1/5.0  (0.3-26.9) | 0/0.0  (0.0-20.0) | 1/5.0  (0.3-26.9) | 1/5.0  (0.3-26.9) | 2/10.0  (1.8-33.1) | 1/5.0  (0.3-26.9) | 2/10.0  (1.8-33.1) |
| **Control** | | 92 | 0/0.0  (95.0 - 100.0) | 0/0.0  (95.0-100.0) | 2/2.2  (91.6-99.6) | 0/0.0  (95.0-100.0) | 2/2.2  (91.6-99.6) | 2/2.2  (91.6-99.6) | 2/2.2  (91.6-99.6) |

**Supplementary Table 3**. Performance of ColoDefence test in detecting different disease states for the subjects who also had CEA, AFP and CA19-9 measurements.

|  | | ***mSEPT9*** | ***mSDC2*** | ***mSEPT9*+*mSDC2*** |
| --- | --- | --- | --- | --- |
| **Sensitivity (95% CI)** | **CRC stage** |  |  |  |
|  | **0+I** | 17.6 (4.7 - 44.2) | 29.4 (11.4 - 26.0) | 35.3 (15.3 - 61.4) |
|  | **II** | 40.0 (24.4 - 57.8) | 20.0 (9.1 - 37.5) | 48.6 (31.7 - 65.7) |
|  | **III** | 52.0 (31.8 - 71.7) | 32.0 (15.7 - 53.6) | 64.0 (42.6 - 81.3) |
|  | **IV** | 79.3 (59.7 - 91.3) | 65.5 (45.7 - 81.4) | 89.7 (71.5 - 97.3) |
|  | **All** | 50.0 (40.2 - 59.8) | 36.8 (27.8 - 46.8) | 61.3 (51.3 - 70.5) |
|  | **AA** | 14.3 (4.7 - 33.6) | 25.0 (11.4 - 45.2) | 39.3 (22.1 - 59.3) |
|  | **CRC + AA** | 42.5 (34.1 - 51.4) | 34.3 (26.5 - 43.1) | 56.7 (47.9 - 65.2) |
|  | **SP** | 5.0 (0.3 - 26.9) | 25.0 (9.6 - 49.4) | 30.0 (12.8 - 54.3) |
| **Specificity (95% CI)** | | 97.8 (91.6 - 99.6) | 92.4 (84.4 - 96.6) | 90.2 (81.8 - 95.2) |
